# Dr. Crawford W. Long, the Discoverer of Anæsthesia. Anæsthesia and Anæsthetics

**Published:** 1879-12

**Authors:** J. J. Caldwell

**Affiliations:** Baltimore, Md.


### DR. CRAWFORD W. LONG, THE DISCOVERER OF ANÆSTHESIA. ANÆSTHESIA AND ANÆSTHETICS.

*Read before the Dental Association of the State of Maryland and the District of Columbia, held in Washington, October 8, 9, and 10, 1878.*

BY DR. J. J. CALDWELL, BALTIMORE, MD.

As to whom the great honor should be awarded of having discovered such a valuable and benign agency as anæsthesia, we can not but coincide with the opinion pronounced by Dr. J. Marion Sims, in the Virginia Medical Monthly, of May, 1877, that the distinguished individual was Dr. Crawford W. Long, of Athens, Georgia, who extirpated a tumor from the neck of James W. Venable, of Jefferson, Jackson county, Georgia, March 30, 1842, while he was competely anæsthetized by the inhalation of sulphuric ether.

As remarked, however, by Dr. Sims, "to each claimant for the honor of discovering anæthesia is due a certain measure of credit." The names of Long, Wells, Morton, and Jackson will doubtless be associated together as co-laborers in the great work, and to these must be added the immortal name of Sir James Y. Simpson, who introduced chloroform and enlarged the domain of anæsthesia.

### A BIOGRAPHICAL SKETCH OF THE WORLD'S GREAT BENEFACTOR.

Dr. Crawford W. Long, the now famous discoverer of surgical anæsthesia, was at the time of his death, June 16, 1879, sixty-three years old.

He was born in Danielsville, Ga., November 1st, 1815. His father, James Long, came with his father, Captain Samuel Long, to Georgia, with his family as a part of a colony of emigrants from Pennsylvania shortly after the revolution of 1776. Captain Long served throughout that eventful struggle; was at the battles of Germantown and Brandywine, and was in the forlorn hope, commanded by Lafayette, at the surrender of Lord Cornwallis at Yorktown, which closed the war.

He was a man of great executive ability, immense driving power and practical intelligence—a born leader and brave, patriotic, modest man, and gave his son James every advantage of education and culture then attainable in the country.

Mr. James Long, the father Dr. Crawford W. Long, was also a man of great ability. He was a merchant and planter, but never ceased to be a student, the law having special attractions for him. He studied medicine in the University of Pennsylvania and was graduated M. D. with high honors from that institution in 1839.

Dr. Long first practiced medicine for many years at Jefferson, Jackson county, where he made his experiments and perfected and demonstracted his great discovery of the anæsthetic properties of ether to annihilate pain under any and all cases of human suffering without endangering or destroying life.

After his graduation, Dr. Long was physician for one year in the New York City hospital. Being passionately fond of surgery and was so successful that he was recommended by the secretary of the navy to be surgeon to a national ship of war, a high honor in those days. He would have accepted this position, but in deference to his father's wishes located and commenced the practice of his profession at Jefferson, Ga., where he performed many difficult and dangerous operations, chiefly the extraction of tumors and the removing of cancers, or other fungoid affections. In 1842 he married Miss Caroline Swain, only child of Geo. Swain, a planter, and brother of David L. Swain, LL. D., governor of North Carolina, and president of the university of that state. When Athens was garrisoned by United States troops after the war he was offered the position of surgeon by the great general and president of the reunited nation, but was unable to take the requisite oath and was commissioned on his merits to the position by Col. Blucher, who informed him that he "wanted no oath from him as his character for honor, truth and justice were well known and established."

Dr, Long was ruined by the war and died struggling to meet engagements and to pay off debts created in New York and other northern cities before the war, in which he became involved unwisely for others. His sons are now his successors in the drug business at Athens, and have paid off over twelve thousands dollars on the whole amount of their noble father's obligations, which is the whole amount.

Dr. Long at his death left two sons and five daughters. The eldest, Miss Fannie, is a self-reliant and highly intelligent young lady, who was the confidant and private secretary of her father, and has all his papers and documents in her keeping.

After her father's death she sought and received employment as a teacher of drawing and painting at Huntsville, Ala. She has been suggested as a member of the committee to select the artist and decide on the position, whether sitting or standing, of the statue of her father; also, as to the material. Her idea being to have the statue placed in the National capitol to be executed in marble, and another duplieate in bronze to be placed in the grounds fronting the proposed new state capitol.

Jenner discovered the method by vaccination of preventing the spread of smallpox. England bestowed upon him high honors. Sir James G. Simpson, of Edinburgh, Scotland, was the first to introduce chloroform as anæsthetic, into general use in 1847. He recieved high honors from England. But we may here state that the medical profession regard sulphuric ether as far preferable to chloroform as an anæsthetic agent. We have no orders of the Cross, of the Garter or titles of nobility to bestow, but we can express our apprectiation of virtue, of merit, of mental achievement by resolution. We can embalm in our hearts the memory of the good and great; we can prepetuate that memory by painting and statuary.

Three agents have been used to produce anæsthesia, namely: nitrous oxide gas, sulphuric ether and chloroform.

For more than two thousand years persons have been put to sleep and had incisions and amputations without pain. But the opiate agents were uncertain and dangerous. For moie than three hundred years sulphuric ether was used by the medical profession without knowing its anæsthetic property.

Dr. C. W. Long concluded to use sulphuric ether in a surgical operation. Living in Jefferson, a very small village in Jackson county, sparsely populated, where little opportunity for surgery was presented, he abided an occasion. On the 30th of March, 1842, he used sulphuric ether upon a Mr. Venable, and excised a tumor from his neck, without the patient being conscious of pain. He made two other experiments in the same year, and other experiments in 1843, as also in subsequent years.

